# Socioeconomic disparities in prevalence and behaviors of smoking in rural Southwest China

**DOI:** 10.1186/s12889-019-7455-0

**Published:** 2019-08-14

**Authors:** Le Cai, Xu-Ming Wang, Lu-Ming Fan, Wen-Long Cui, Allison Rabkin Golden

**Affiliations:** 1 School of Public Health, Kunming Medical University, 1168 Yu Hua Street Chun Rong Road, Cheng Gong New City, Kunming, 650500 China; 2 The First Affiliated Hospital of Kunming Medical University, Western Renmin Road, Kunming, 650032 China

**Keywords:** Smoking, Behaviors, Socioeconomic status, China

## Abstract

**Background:**

This study examines how prevalence and behaviors of smoking differ by socioeconomic status among rural southwest Chinese adults.

**Methods:**

A cross-sectional survey was conducted including 7743 adults aged ≥35 years in rural regions of Yunnan Province, China from 2016 to 2018. Information on individual socioeconomic status (SES), ethnicity, and self-reported smoking behaviors was collected utilizing a standardized questionnaire. The individual socioeconomic position (SEP) index was constructed using principal component analysis. Multivariate logistic regression models were used to analyze the association between individual SES variables and the prevalence and behaviors of smoking.

**Results:**

In the study population, the overall prevalence rate of current smokers was 33.5%. Males had a markedly higher prevalence of current smokers than females (62.6% vs. 4.8%, *P* < 0.01). Of these smokers, 74.5% began smoking during adolescence, 88.8% had never attempted to quit smoking, and 81.1% reported smoking in public places. Ethnic minority participants and those with low levels of education and/or low SEP were more likely to use tobacco as well as more likely to start smoking, and regularly smoke, during adolescence (*P* < 0.01). Participants with poor access to medical services had a higher prevalence of current smoking than their counterparts (*P* < 0.01). Among current smokers, Han ethnicity, good access to medical services, and high SEP were positively associated with the probability of having attempted to quit smoking at least once, while a high level of education and high SEP were negatively associated with the probability of smoking in public places.

**Conclusions:**

Disparities in prevalence and behaviors of smoking exist across a diversity of indicators of individual SES in rural southwest China. Future tobacco cessation interventions should focus on men, ethnic minorities, and those with low education levels, poor access to medical services, and low SEP.

## Introduction

Tobacco smoking causes many preventable diseases and its impact on premature death is a major concern worldwide: the World Health Organization (WHO) reports that smoking accounts for almost 6 million deaths globally each year [1]. China is both the world’s largest tobacco manufacturer and consumer, accounting for one-third of the world’s tobacco production and consumption [2]; it is home to 300 million smokers and 740 million non-smokers exposed to secondhand smoke [3, 4]. In turn, China has experienced a large-scale increase of smoking-related chronic disease, creating an immense public health challenge and, consequently, a significant financial burden, especially in rural communities [5]. The serious dangers that smoking poses to health are evident. Yet, implementing effective tobacco control programs has been challenging, particularly in rural, tobacco cultivating regions.

Growing evidence both in developed and developing countries suggests that smoking behaviors are associated with socioeconomic status (SES). Those with less education and lower incomes are more likely to smoke [6–8]. Prior research also indicates that early smoking initiation is negatively correlated with educational attainment, and people with low SES status and lower incomes are less likely to be successful when they attempt to quit smoking [9–11]. In China, several studies have investigated the relationship between patterns of smoking and SES factors [5, 12], revealing an inverse relationship between education and smoking. Higher educational attainment was also associated with lower likelihood of smoking behaviors among the elderly population in China [13]. China is a multi-ethnic country with 56 state-recognized ethnicities. The Han ethnicity makes up the majority of the population (> 92%) while the remaining 55 ethnic groups are classified as national minorities. Seventy percent of China’s population lives in the countryside. However, due to a lack of research detailing the relationship between individual socioeconomic factors and smoking behaviors, socioeconomic disparities in smoking initiation, quit attempts, and level of tobacco consumption remain poorly understood, particularly in rural populations and ethnic minority groups, the subgroups with the highest prevalence of tobacco use in China [14]. Due to the fact that SES factors are an important determinant of smoking behaviors, a better understanding of SES determinants of prevalence and behaviors of smoking is vital for facilitating the implementation of tailored tobacco control and prevention programs in China. Thus, research that can detail socioeconomic disparities in smoking behaviors holds particular importance.

The aim of this study is to address this research gap by uncovering how prevalence and behaviors of smoking– including current smoking status, level of daily smoking, age of smoking initiation, age of regular smoking, quit attempts, and location of smoking behaviors— differ across socioeconomic groups among southwest China’s rural adult population.

## Methods

### Study area and population

Yunnan Province is located in southwestern China. It has 129 official counties, among which rural communities account for 70.5% of total population, and is a production and consumption hub for tobacco products in China, with the tobacco industry supporting a large portion of the local economy. Roughly 70% of Yunnan Province’s annual tax revenue is collected from the tobacco industry. Yunnan has an obvious price advantage in tobacco and tobacco products competition compared to other provinces in China. Yunnan is also home to the country’s largest concentration of ethnic minorities, with 25 ethnic minority groups living in the province. Over the last two decades, China has implemented tobacco control legislation aimed at curbing tobacco use. Namely, China officially ratified the WHO Framework Convention on Tobacco Control (FCTC) in October 2005, which came into force in January 2006. Currently, China does not have a comprehensive tobacco control law at the national level. However, in recent years, Yunnan Province has passed local regulations and made efforts to implement comprehensive bans on smoking in all indoor public places, workplaces including hospitals and schools, and public transportation. However, progress on controlling tobacco use has not moved quickly. Problems with implementation and enforcement remain in Yunnan, with advertising of tobacco products not fully restricted, and smoking during adolescence still a serious public health issue in rural and ethnic minority regions.

In this study, a community-based cross-sectional survey was conducted in three rural areas of Yunnan Province from January 16, 2016 to January 28, 2018.

### Sampling

The present study used a multi-stage stratified random sampling technique designed to select a representative sample of participants aged 35 years and over. In the first stage, 129 counties were divided into three categories according to per capita gross domestic product (GDP): economically advantaged, economically average, and economically disadvantaged. From each of these three categories one township was then randomly selected, for a total of 9 townships. In the second stage, three villages were chosen by probability proportional to size (PPS) within each of the 9 townships. In the third stage, simple random sampling was conducted to select the sample subjects from a village based on a list of individuals aged ≥35 years obtained from each selected village’s leadership committee.

### Data collection and measurement

Each participant who signed an informed consent form was personally interviewed by trained interviewers using a pre-tested and structured questionnaire. The questionnaire included questions about individual SES, ethnicity, and self-reported smoking and drinking habits.

### Definitions

Current smokers were defined as individuals who had both smoked at least 100 cigarettes over their lifetime and reported smoking any tobacco product on a daily basis during the survey period [15]. Age of smoking initiation was defined as the age at which a person first used any kind of tobacco product. Amount of tobacco consumption was defined as the number of cigarettes an individual smoked per day. Current drinkers were defined as drinking alcohol regularly on 12 or more days during the previous 12 months [16]. Ethnic minority status was defined as having a different religion, culture, or language from that of the majority Han population. Illiterate was defined as the inability of a person aged 15 years and over to read and write, with understanding, a short simple sentence on his or her everyday life.

### Measurement of socioeconomic status

Data gathered on individual socioeconomic indicators were organized into three categories: household assets, education level, and access to medical services. Household assets included measurements of annual household income, the existence of a toilet and running water in the house, and housing facility materials. Household assets were classified into two categories: rich and poor. Rich was defined as annual income ≥ $850 US and a brick or concrete house containing both tap water and a toilet. Poor was defined as household income <$850 US and an adobe or stone house containing neither tap water nor a toilet. Access to medical services was defined as good when walking time to the nearest village hospital was less than 30 min and poor when walking time to the nearest village hospital exceeded 30 min.

### Statistical analysis

Descriptive analyses were used to calculate absolute and relative frequencies (%). Categorical variables were presented as counts and percentages. Age-adjusted prevalence rates of current smoking were calculated by a direct method using China’s adult population aged 35 years and over, with 2010 as the standard population. A chi-squared test was used to compare categorical variables. Multivariate logistic regression models were used to analyze the association between individual SES variables and the prevalence and behaviors of smoking, adjusted by age, sex, ethnicity, and current drinking status. Associations were expressed as odds ratios and 95% confidence interval (CI).

A composite index was used as a proxy for individual socioeconomic position (SEP), and principal components analysis (PCA) was used to construct the SEP index. The SEP for this estimation included three socioeconomic indicators— household assets, educational level, and access to medical services— based on an estimated Pearson correlation index greater than 0.5. PCA was performed to determine the number of potential components that would define an index or indexes of individual SEP. Bartlett’s test of sphericity was used to assess whether the correlation between indicators was adequate based on a criterion of *p* < 0.0001. The Kaiser-Meyer-Olkin (KMO) statistic was used to measure sample adequacy based on a criterion of ≥0.7. A threshold of eigenvalues greater than one was used as the criterion for the extraction of principal components. All statistical significance decisions were based on two-tailed *P* values. All data analyses were conducted with SPSS 22.0 software.

## Results

A total of 8100 individuals aged ≥35 years were invited to participate in the study. Of these, 7743 agreed to participate (a response rate of 95.6%).

Bartlett’s test of sphericity was significant (*P* < 0.0001) and the KMO measure was satisfactory (0.71), indicating that correlations between variables were sufficiently large to conduct a PCA. The results of PCA analysis indicated that only the first component had an eigenvalue greater than one, explaining 58.1% of the total variance; therefore, only one component was used to define the SEP index. The SEP index was then further divided into two categories (high and low) with the median value as the cut off.

Demographic characteristics of the participants are summarized in Table 1. In total, 3832 males (49.5%) and 3911 females (50.5%) participated. The percentage of ethnic minority participants was 39.7%, and illiterate rate was 21.8%. Male participants had higher SES status, higher education levels, household assets, and access to medical services than female participants (*P* < 0.05).

**Table 1:** Demographic characteristics of the study population

Characteristic	Male *n* = 3832 (%)	Female *n* = 3911 (%)	All *n* = 7743 (%)
Age			
35–44 years	961 (25.1)	957 (24.5)	1918 (24.8)
45–54 years	1025 (26.1)	1098 (28.1)	2123 (27.4)
55–64 years	888 (23.2)	993 (25.4)	1881 (24.3)
≥ 65 years	958 (25.0)	863 (22.1)	1821 (23.5)
Ethnicity			
Han	2310 (60.3)	2277 (58.2)	4587 (59.2)
Minority	1522 (39.7)	1634 (41.8)	3156 (40.8)
Level of education			
Illiterate	654 (17.1)	1037 (26.5)**	1691 (21.8)
Primary (grade 1–6) or higher	3178 (82.9)	2874 (73.5)	6052 (78.2)
Household assets			
Good	2597 (67.8)	2964 (75.8)**	5561 (71.8)
Poor	1235 (32.2)	947 (24.2)	2182 (28.2)
Access to medical services			
Good	2933 (76.5)	2892 (73.9)*	5825 (75.2)
Poor	899 (23.5)	1019 (26.1)	1918 (24.8)
SES status			
High	1833 (47.8)	1345 (34.4)	3178 (41.0)
Low	1999 (52.2)	2566 (65.6)**	4565 (59.0)

Chi-squared test, * *p* < 0.05, ** *p* < 0.01

Table 2 shows the age-standardized prevalence of current smokers by age, sex, and ethnicity among the study participants. The overall prevalence rate of current smokers was 33.5% (62.6% for males, and 4.8% for females). Males had significantly higher prevalence of current smokers than females (*P* < 0.01). Prevalence of current smokers decreased with age, peaking at 35.3% in the total population aged 35–44 years. The ethnic minority population had a higher prevalence of current smokers than the Han majority population, both for males and females (*P* < 0.01).

**Table 2:** Age-standardized prevalence of current smoking by sex, age, and ethnicity in Yunnan Province, China

Characteristic	Men n (%)	Women n (%)	All n (%)
Age			
35–44 years	635 (69.2)**	29 (3.3)**	664 (35.3)*
45–54 years	707 (66.3)	52 (4.9)	759 (34.6)
55–64 years	559 (62.7)	62 (6.0)	621 (33.1)
≥ 65 years	504 (53.8)	49 (6.2)	553 (30.0)
Ethnicity			
Han	1378 (60.3)	25 (1.2)	1403 (30.6)
Minority	1027 (66.8)*	167 (10.1)**	1194 (37.8)**
All	2405 (62.6)**	192 (4.8)	2597 (33.5)

* *p* < 0.05, ** *p* < 0.01

Table 3 presents smoking behaviors by ethnicity among current smokers. Filtered cigarettes were the most popular method of smoking tobacco, comprising over 75.0% of all types of tobacco consumed. 74.5% of current smokers began smoking during adolescence, with 20.1% of smokers reporting regular smoking during adolescence. In the 12 months prior to the survey, most tobacco users (88.8%) had made no attempt to quit smoking. 73.6% of smokers reported having smoked in public places in the 7 days prior to the survey.

**Table 3:** Smoking behaviors by ethnicity among current smokers in rural areas of Yunnan Province, China

Variable	Han majority n (%)	Minorities n (%)	All n (%)
Use of various forms of tobacco			
Filtered cigarettes	999 (77.2)	894 (75.1)	1893 (76.2)
Hookah/Water pipe	195 (15.1)	16 (1.3)	211 (8.5)
Chewing tobacco	1 (0.1)	181 (15.2)	182 (7.3)
Hand-rolled cigarettes	71 (5.5)	147 (12.3)	218 (8.8)
Unfiltered cigarettes	115 (8.9)	46 (3.9)	161 (6.5)
Pipe tobacco	32 (2.5)	4 (0.3)	36 (1.4)
Age of smoking initiation			
< 18 years	917 (64.0) **	961 (78.8)	1878 (74.5)
> =18 years	413 (36.0)	231 (21.2)	644 (25.5)
Age of regular smoking			
< 18 years	162 (12.2)**	345 (28.9)	507 (20.1)
> =18 years	1168 (87.8)	847 (71.1)	2015 (79.9)
Number of cigarettes per day			
< 10	265 (22.5)*	335 (28.2)	600 (25.3)
> =10	913 (77.5)	854 (71.8)	1767 (74.7)
Previous quit attempts			
No attempt	1139 (81.2)**	1168 (97.8)	2307 (88.8)
At least one attempt in previous 12 months	264 (18.8)	26 (2.2)	290 (11.2)
Location of smoking in previous 7 days			
Public spaces (schools, hospitals, etc.)	748 (53.3)**	1164 (97.5)	1912 (73.6)
Home	655 (46.7)	30 (2.5)	685 (26.4)

* *p* < 0.05, ** *p* < 0.01

There were significant differences in smoking behaviors by ethnicity. After filtered cigarette smoking, hookah/water pipes were the second most popular form of tobacco consumption in the Han majority population, followed by unfiltered cigarettes. In contrast, chewing tobacco was the second most popular form of tobacco consumption in the ethnic minority population, followed by hand-rolled cigarettes. Compared to the Han majority population, ethnic minorities had both a higher proportion of smokers who initiated smoking during adolescence as well as a higher proportion of smokers who regularly smoked during adolescence (*P* < 0.01). Among current smokers, whereas the prevalence of smoking more than 10 cigarettes daily was higher in the Han majority population than in the ethnic minority population (*P* < 0.05), ethnic minorities had a higher proportion of smokers who had never attempted to quit smoking and a higher proportion of smokers who smoked in public places than the Han majority (*P* < 0.01).

Table 4 indicates the results of multivariate logistic regression analysis of prevalence and behaviors of smoking by individual SES after adjusting for age, sex, ethnicity, and current drinking status. Individuals with lower levels of education, poor access to medical services, and lower SEP were more likely to consume tobacco, whereas current smokers with rich household assets and a higher SEP had a higher risk of smoking more than 10 cigarettes daily. Individuals with low education levels and low SEP were more likely to initiate smoking and regularly smoke during adolescence. Among current smokers, access to medical services and SEP were positively associated with the probability of having attempted to quit smoking at least once. Current smokers with higher levels of education and higher SEP had a lower probability of smoking in public places.

**Table 4:** Logistic regression for prevalence and behaviors of smoking by socioeconomic status

Variable	Current smoking (reference: no)		Amount of smoking per day (reference: < 10 cigarettes)		Age of smoking initiation (reference: < 18 years)		Age of regular smoking (reference: < 18 years)		Previous quit attempts (reference: no)		Location of smoking (reference: home)	
	Adjusted odds ratio^a^	95% CI	Adjusted odds ratio^a^	95% CI	Adjusted odds ratio^a^	95% CI	Adjusted odds ratio^a^	95% CI	Adjusted odds ratio^a^	95% CI	Adjusted odds ratio^a^	95% CI
Level of education (reference: Illiterate)	0.32**	(0.26, 0.38)	1.27	(0.97, 1.67)	1.78**	(1.41, 2.25)	1.61**	(1.25, 2.07)	0.79	(0.53, 1.18)	0.72*	(0.53, 0.96)
Household assets (reference: Poor)	0.86	(0.71, 1.03)	1.57**	(1.23, 2.03)	0.67	(0.49, 1.02)	0.68	(0.48, 1.02)	0.92	(0.65, 1.29)	0.83	(0.62, 1.11)
Access to medical services (reference: Poor)	0.82**	(0.71, 0.95)	1.14	(0.82, 1.59)	0.73	(0.58, 1.01)	0.95	(0.75, 1.21)	1.02*	(1.01, 1.32)	0.81	(0.63, 1.03)
SES status (reference: Low)	0.84**	(0.74, 0.95)	1.39**	(1.11, 1.73)	1.26*	(1.05, 1.41)	1.36*	(1.07, 1.90)	1.68**	(1.18, 2.40)	0.85**	(0.76, 0.97)

* *p* < 0.05, ** *p* < 0.01

^a^ adjusted for age, sex, ethnicity, and current drinking status

## Discussion

This cross-sectional study reveals a high prevalence rate of current smokers, a high prevalence of smoking initiation during adolescence, and a low rate of attempts to quit smoking in rural southwest China. This study also demonstrates that there are significant relationships between SES variables and prevalence and behaviors of smoking.

In this study, the prevalence of current smoking in both sexes (male 62.6%, female 4.8%) was higher than the prevalence rate observed in urban Chinese populations (male 58.4%, female 3.4%) [17] as well as that observed in other Asian countries [4, 18]. However, the rate of smokers who had previously attempted to quit smoking (11.2%) was much lower than reported by the 2010 National China Smoking Survey (17.3%) and that in other Asian countries (59.9%) [19]. Furthermore, men had a remarkably higher prevalence of smoking than women. Such sex differences in smoking are well recognized worldwide: studies in Asia (including China) and Western countries have established many more men than women smoke [17–19]. Our findings indicate smoking is especially prevalent among men in rural southwest China, and robust smoking cessation programs are urgently needed in rural communities.

The present study found that ethnic minorities had a higher prevalence of current smokers for both males and females and a much lower rate of quit attempts than their Han counterparts. Furthermore, the rate of smoking in public places was also higher in ethnic minorities than in the Han majority population. This may largely be explained by the higher illiteracy rate in the ethnic minority population (32.0%) compared to the Han majority (14.5%), as lower levels of education have a strong effect on smoking habits [13, 20]. The findings suggest tobacco control interventions are particularly needed in rural minority communities.

Several previous Chinese studies indicate that smoking is a serious problem among Chinese teenagers [14, 21]. Our study is consistent with this prior research, finding that 74.5% of smokers began smoking during adolescence. Moreover, ethnic minorities had an earlier age of smoking initiation and of regular smoking than their Han counterparts. These findings underscore an urgent need to strengthen adolescent tobacco control programs, particularly among ethnic minorities in rural China.

In China, traditions, heritage, culture, and lifestyles differ by ethnic group. Previous studies indicated that there are pronounced ethnic variations in prevalence of smoking and form of tobacco use. This possibly results from the strong collective social life in many ethnic minority communities, as well as differing cultures and shared environmental influences related to smoking behaviors [16, 22, 23]. Our study found that filtered cigarettes were the most popular form of tobacco used in rural southwest China, a finding consistent with previous Chinese studies [14]. However, chewing tobacco was also common among ethnic minorities. Our findings thus demonstrate that ethnicity plays an important role in shaping smoking habits, and culturally appropriate tobacco control interventions could help reduce tobacco product consumption in rural China.

Some previous Chinese studies indicated that public places have the highest secondhand smoke exposure in China [5, 24]. In the present study, the rate of smoking in public places (73.6%) was much higher than found in a previous Chinese study examining 21 Chinese cities (41.2%) [24]. Moreover, individuals with lower levels of education and lower SEP had a higher probability of smoking in public places. These results suggest it is essential to implement smoke-free regulations to protect people from secondhand smoking exposure in rural China, and underscore that efforts to restrict smoking in public places should focus on people with lower education levels and lower SEP.

Lower educational levels are associated with higher risks of smoking [5, 6, 12, 13]. Moreover, prior research has indicated that the highest proportion of smokers in low- and middle-income countries have low SES [25]. Our results are in accordance with these studies. Furthermore, the present study also found that individuals with poor access to medical services are more likely to be current smokers. This is possibly due to the fact that better access to medical services is associated with better access to health related knowledge as well as a higher awareness of tobacco hazards. Thus, our findings suggest that future community-based tobacco control intervention programs need to provide education on the harmful effects of tobacco use, particularly to those with low education levels and low SEP in rural China. They also suggest improved access to healthcare is crucial to controlling smoking.

While several studies from both developing and developed countries have demonstrated that individuals with lower household incomes have a higher risk of smoking [7, 26, 27], our study showed no association between household assets and smoking. This result is in line with a previous Chinese study [13]. However, the present study did find that rich household assets and higher SEP were associated with a higher risk of smoking more than 10 cigarettes daily among current smokers. This possibly results from the fact that smokers with a higher household income and SEP are better able to afford tobacco products. Further investigation is needed to examine the exact nature of the association between income and smoking behaviors.

This study also found that access to medical services was positively associated with the probability of having attempted to quit smoking at least once. This result can possibly be attributed to increased ability to utilize health services and greater knowledge about the harmful effects of tobacco use among these populations. Moreover, higher individual SEP showed a positive association with attempting to quit smoking. A positive relationship between individual SEP and smoking cessation was also observed in a study of a western population [28]. Perhaps those with higher SEP tend to have a healthier lifestyle and therefore consume less tobacco. The findings in this way also indicate that socioeconomic inequality affects behaviors related to smoking cessation.

The following limitations of the present study should be noted. First, smoking behaviors were self-reported, and the lack of validation of smoking status with nicotine testing may underestimate the prevalence of smoking. Second, the present findings were based on a random sampling of three counties, limiting their generalizability.

In conclusion, the present study indicates smoking is serious public health problem posing a major challenge for tobacco control and smoking prevention in rural southwest China, with ethnicity and socioeconomic factors playing an important role in influencing both prevalence and behaviors of smoking. Addressing the socioeconomic determinants of smoking behaviors and providing culturally appropriate tobacco control interventions could help minimize disparities in tobacco use and smoking behaviors and thereby promote public health.

## Data Availability

The datasets used and/or analysed during the current study is available from the corresponding author on reasonable request.

## Abbreviations

CI: Confidence Interval
FCTC: Framework Convention on Tobacco Control
GDP: Gross Domestic Product
KMO: Kaiser-Meyer-Olkin
PCA: Principal Components Analysis
PPS: Probability Proportional to Size
SEP: Socioeconomic Position
SES: Socioeconomic Status
WHO: World Health Organization
